# Food allergens labelling in Argentina: a global approach

**DOI:** 10.1186/2045-7022-1-S1-P17

**Published:** 2011-08-12

**Authors:** Maria Cristina Lopez, Claudia Gonzalez, Larua Lopez

**Affiliations:** 1 National Institute of Industrial Tecnology, Cereals and Oilseeds Center, San Martin, Argentina; 2 National Institute of Agricultural Technology, Buenos Aires, Argentina; 3 Buenos Aires University, School of Pharmacy and Biochemistry, Buenos Aires, Argentina

## 

Food allergy is a public health concern in almost all over the world. Although allergic responses to food allergens vary markedly due to geographical differences, the lists of allergenic ingredients enshrined in different legislations are based on the original Codex recommendations with some regional variations. The patterns and prevalence of food allergy in Argentina -according to a survey carried out by the Argentine Allergy and Clinical Immunology Association in 2007- show that 75% of the population suffered from a "potential" food allergy to one of the "big eight" listed in the Codex Standard.

Latin American countries are incorporating the allergens declaration in the ingredients list of prepackaged foods. Even though most of them follow Codex Standard, there are exceptions. One of them is from Argentine legislation, which came into force last September. In general, the legislation follows the Codex list and adds some of the EU exceptions, however it includes tartrazine recommendations. Regarding the permission for the use of precautionary labelings, its use is explicitly forbidden.

Although, different legislations do not include potential cross contamination and there are currently no international agreements in relation to regulatory or advisory limits, precautionary labeling is used worldwide or at least it is not explicitly forbidden.

The aim of this presentation is to show the situations that have aroused in Argentina after the application of this new legislation and the collaborative actions carried out by the Platform of Food Allergens and the Argentinean government. These issues are related to the food industry and to allergic patients, which might become a future matter of concern for international trade.

